# Comprehensive proteome and phosphoproteome profiling shows negligible influence of RNAlater on protein abundance and phosphorylation

**DOI:** 10.1186/s12014-019-9239-z

**Published:** 2019-04-25

**Authors:** Jingi Bae, Su-Jin Kim, Seung-Eun Lee, Wooil Kwon, Hongbeom Kim, Youngmin Han, Jin-Young Jang, Min-Sik Kim, Sang-Won Lee

**Affiliations:** 1 Department of Chemistry, Center for Proteogenome Research, Korea University, Seoul, 136-701 Republic of Korea; 2 Department of Biomedical Science and Technology, Kyung Hee Medical Science Research Institute, Kyung Hee University, Seoul, Republic of Korea; 3 Department of Surgery and Cancer Research Institute, Seoul National University College of Medicine, Seoul, Republic of Korea; 4 Department of New Biology, DGIST, Daegu, 42988 Republic of Korea

## Abstract

Certain tumors such as pancreatic ductal adenocarcinoma (PDAC) are known to contain a variety of hydrolytic enzymes including RNases and proteases that may lead to degradation of RNA and proteins during sample processing. For such tumor tissues with RNA instability, RNAlater containing a high concentration of quaternary ammonium sulfates that denature RNA-hydrolyzing enzymes is often used to protect RNAs from hydrolysis. Although a few studies have been carried out to determine the effect of RNAlater on DNA and RNA, whether RNAlater influences the proteome and phosphoproteome is largely unknown. In this study we carried out a systematic and comprehensive analysis of the effect of RNAlater on the proteome and phosphoproteome using high-resolution mass spectrometry. PDAC tissues from three patients were individually pulverized and the tissue powders of each patient were divided into two portions, one of which was incubated in RNAlater at 4 °C for 24 h (RNAlater tissue) while the other was kept at – 80 °C (frozen tissue). Comprehensive quantitative profiling experiments on the RNAlater tissues and the frozen tissues resulted in the identification of 99,136 distinct peptides of 8803 protein groups and 17,345 phosphopeptides of 16,436 phosphosites. The data exhibited no significant quantitative changes in both proteins and phosphorylation between the RNAlater tissues and the frozen tissue. In addition, the phosphoproteome data showed heterogeneously activated pathways among the three patients that were not altered by RNAlater. These results indicate that the tissue preservation method using RNAlater can be effectively used on PDAC tissues for proteogenomic studies where preservation of intact DNA, RNA and proteins is prerequisite. Data from this study are available via ProteomeXchange with the identifier PXD010710.

**Electronic supplementary material:**

The online version of this article (10.1186/s12014-019-9239-z) contains supplementary material, which is available to authorized users.

## Background

Analysis of disease tissues is the first step toward understanding the underlying biology of the disease. Since the nature of larger molecules such as DNA, RNA, and proteins in tissues may vary according to their environment, methods of storing clinical tissue samples have to meet a standard operating procedure to minimize pre-analytical variation [1]. There are a few widely-used tissue storage methods for the purpose of preservation, including snap-freezing, formalin fixation, and RNAlater [2–4]. Snap-freezing involves the rapid cooling of clinical samples in liquid nitrogen, which is the quickest way to preserve all molecules in the samples and is considered the best method of storing clinical samples as long as the samples are placed into liquid nitrogen immediately after collection with a short and controlled ischemic time. However, in practice this simple procedure can be difficult to follow in certain clinical settings of surgery. More importantly, the major focus of doctors in operating rooms is to treat the patient properly rather than to collect tissue samples. Conventional formalin fixation is a relatively easier process that can preserve tissue architecture and is generally combined with embedding in paraffin [5].

As an alternative method, RNAlater can stabilize RNAs by inhibiting RNases in the samples since it contains a high concentration of quaternary ammonium sulfates and cesium sulfate [6–8]. RNAlater has become the widely adopted reagent for storing RNAs with the aim of studying gene expression. Although tissues can be stored in a – 80 °C freezer after snap-freezing and/or RNAlater treatment, some tissues that are only available in tiny amounts cannot be easily aliquoted and stored in multiple ways, limiting experimental exploration. In such cases, RNAlater may be the method of choice for tissue storage for subsequent molecular analysis of gene expression and mutation searches. RNAlater treatment was demonstrated to help obtain high-quality RNAs from human pancreas tissues [9], bacterial DNAs [10] and biological molecules [11]. A few studies have explored the effect of RNAlater on biomolecules such as DNA, RNA, and proteins. For example, Kruse et al. [12] reported that a small part of the transcriptome and proteome of *Arabidopsis thaliana* was slightly altered with RNAlater. Bennike et al. [13] also reported only minor quantitative changes in global tissue proteomes caused by RNAlater. Although RNAlater is generally believed not to affect the global proteome, the step of tissue incubation in RNAlater solution overnight at 4 °C may induce ischemia, which is known to change protein phosphorylation [14].

In this study, we carried out in-depth systematic analysis of proteomes and phosphoproteomes of pancreatic ductal adenocarcinoma (PDAC) tissues treated with RNAlater. Tumor tissues from three patients were used to prepare tryptic peptides that were labeled with 6-plex TMT reagent for quantitative mass spectrometry. Labeled peptides were fractionated into 24 fractions for global proteomic analysis and 12 fractions for phosphoproteomic analysis. The global proteomic profiling identified 98,223 unmodified peptides of 8803 protein groups, while phosphoproteomic analysis resulted in the identification of 16,436 phosphosites. From this dataset, we found no significant changes in the abundance of proteins and phosphorylations due to RNAlater. Our result indicates that tissues stored in RNAlater can be used not only for transcriptomics but also for proteomics and phosphoproteomics.

## Methods

### Tissue collection

Pancreatic ductal adenocarcinoma tissue samples from three patients were collected at SNUH. This study was approved by the SNUH institutional review board (IRB). All surgical tissues were carefully and quickly washed in saline to remove blood components and immersed in liquid nitrogen within 30 min and then stored at – 80 °C until the next step. Details of the clinicopathological features of the three patients are available in Additional file 1: Table S1. All patients were at the same stage with similar tumor size and disease free survivals.

### Tissue pulverization

Fresh frozen surgical tumor tissues were individually cryopulverized using a Cryoprep device (CP02, Covaris) as described previously [15]. Briefly, each tissue piece (30–130 mg in wet tissue weight) was placed in a cryovial (Covaris, 430487) on dry ice and then transferred to a Covaris tissue bag (TT5 extra thick, Covaris), which was immersed in liquid nitrogen for 30 s. The tissue was pulverized using different impact levels depending on the total weight of the tissue (< 50 mg: impact level 2; 50–150 mg: level 3).

### RNAlater treatment

Each tissue powder was divided into two portions: one half for fresh frozen storage and the other half for storage in RNAlater (Ambion, Austin, TX, USA). Fresh frozen samples were stored for 24 h at – 80 °C and briefly immersed in RNAlater before lysis. For storage in RNAlater, the volume of RNAlater added to fresh frozen powders depended on the weight of the tissue (< 20 mg tissue powder, 100 μL RNAlater; > 50 mg tissue powder: 200 μL RNAlater). RNAlater-treated samples were stored for 24 h at 4 °C.

### Peptide preparation

In order to remove RNAlater, 2× volume of Tris-HCl pH 7.6 buffer was added to dilute RNAlater. After centrifugation at 14,000 g and 4 °C for 2 min (5810 R, Eppendorf), the supernatant was carefully removed. The dilution followed by centrifugation was repeated twice. After removal of RNAlater, the tissue powder sample was mixed with lysis buffer (4% SDS, 0.1 M Tris-HCl pH 7.6, and phosphatase inhibitor [PhosSTOP, Roche]) and lysed by sonication using a probe sonicator (Q55 Sonicator, Qsonica) five times for 30 s on ice until there were no remaining tissue pieces. The homogenate was centrifuged at 16,000*g* and 20 °C for 10 min and the supernatant was transferred to a new tube. Protein concentration was measured using the BCA protein assay (BCA Protein Assay Kit, Pierce).

The lysate was digested using the FASP protocol as described previously [15]. The proteins were reduced in SDT buffer (4% SDS in 0.1 M Tris-HCl, pH 7.6, and 0.1 M DTT) at 37 °C for 45 min with shaking at 300 rpm and boiled for 10 min at 95 °C on a thermomixer (Comfort, Eppendorf). The protein sample was then transferred to a membrane filter (YM-30, Millipore Corporation), in which it was mixed with 200 μL of 8 M urea (in 0.1 M Tris-HCl, pH 8.5). The protein sample on the membrane filter was centrifuged at 14,000*g* and 20 °C for 60 min three times to remove SDS. Subsequently, 100 μL of 0.05 M iodoacetamide in 8 M urea was added for 25 min at room temperature in the dark to alkylate free thiol groups in proteins. The protein samples on the membrane filters were diluted with 200 μL of 8 M urea and concentrated four times. Finally, 100 μL of 50 mM ammonium bicarbonate, pH 8.0, was added to the filter, followed by two rounds of centrifugation at 14,000*g* and 20 °C for 30 min. Trypsin (Promega; Madison; WI) was added to the filter unit at an enzyme to protein ratio of 1:50 (w/w) and the proteins were digested at 37 °C overnight. The second digestion was carried out with trypsin (1:100 ratio) at 37 °C for 6 h. The resulting peptides were eluted by centrifugation at 14,000*g* and 20 °C for 30 min. The filter was rinsed with 60 μL of 50 mM ammonium bicarbonate and centrifuged at 14,000*g* and 20 °C for 20 min. The peptides in the eluents were combined, dried, and kept at − 80 °C until the subsequent TMT labeling.

### TMT labeling of peptides

Peptides were labeled using 6-plex TMT reagent (Thermo Scientific, Rockford, IL). Peptide samples (500 μg each) were labeled with 126, 127, 128, 129, 130, and 131 TMT reagents, respectively. The chemical labeling of peptides with TMT was carried out according to the manufacturer’s instructions (Thermo Scientific). Briefly, the prepared TMT reagent was transferred to the peptide sample and the mixture was vortexed briefly and incubated for 1 h at room temperature in a thermomixer. Excess reagents were quenched by addition of 8 μL of 5% hydroxylamine (Sigma Aldrich) and incubation for 20 min at room temperature. Each set of six TMT-labeled peptide samples was pooled and dried using vacuum centrifugation and subjected to mid-pH reverse-phase liquid chromatography fractionation (mRP fractionation).

### Mid pH reverse-phase liquid chromatography fractionation

The pooled TMT-labeled peptide sample was subjected to mRP fractionation using Agilent 1260 Infinity HPLC system (Agilent, Palo Alto, CA) [16]. A Xbridge C18 analytical column (4.6 mm × 250 mm, 130 Å, 5 um) and a guard column (4.6 mm × 20 mm, 130 Å, 5 um) were used for peptide separation. Solvents A and B were 10 mM triethylammonium bicarbonate (TEAB) in water (pH 7.5) and 10 mM TEAB in 90% acetonitrile (ACN, pH 7.5), respectively. Peptide fractionation was accomplished using a 115 min gradient at a flow rate of 500 μL/min as follows: 0% solvent B for 10 min, from 0 to 5% solvent B over 10 min, from 5 to 35% solvent B over 60 min, from 35 to 70% solvent B over 15 min, 70% solvent B for 10 min, from 70 to 0% solvent B over 10 min. A total of 96 fractions were collected every minute from 15 to 110 min and were pooled into 24 non-contiguously concatenated peptide fractions (i.e., #1–#25–#49–#73, #2–#26–#50–#74, …, #24–#48–#72–#96). The resultant 24 fractions were dried and stored at – 80 °C.

### Phosphopeptide enrichment

Before phosphopeptide enrichment, the 24 peptide fractions were further concatenated into 12 fractions by pooling two adjacent fractions (i.e., #1–#2, #3–#4, …, #23–#24 fraction). Phosphopeptides were enriched as described previously [17]. Briefly, IMAC beads were prepared from Ni-NTA magnetic beads (36113, Qiagen GmbH). The Ni-NTA bead slurry was washed with deionized water and then reacted with 100 mM EDTA (pH 8.0) by gentle mixing for 30 min on an end-over-end rotator (SB3, Stuart). The beads were then reacted with freshly prepared 10 mM aqueous FeCl_3_ solution for 30 min with end-over-end rotation. The resultant Fe^3+^-NTA beads were washed, resuspended in 1:1:1 ACN/MeOH/0.01% acetic acid, and aliquoted into 12 microcentrifuge tubes, each of which contained 100 μL bead solution. Fe^3+^-NTA beads of each tube were washed with 500 μL binding buffer (80% ACN/0.1% TFA). Each of the TMT labeled peptide samples (approximately 250 μg each) of the 12 fractions was resuspended in 500 μL of binding buffer and then transferred to a tube of the aliquoted beads. The binding reactions proceeded for 30 min with end-over-end rotation and the reacted beads were washed four times with binding buffer. Finally, the bound phosphopeptides were eluted from the beads by incubation in 125 μL of 1:1 ACN/2.5% ammonia in 2 mM phosphate buffer (pH 10) for 1 min 30 s. The eluted phosphopeptides were acidified immediately with 10% TFA to pH 3.5–4.0 before vacuum drying.

### LC-MS/MS experiments

TMT-labeled 24 fractions of global peptide samples and 12 fractions of phosphopeptide samples were analyzed using a Q Exactive orbitrap mass spectrometer (Thermo Fisher Scientific, Bremen, Germany) coupled with a modified nanoACQUITY LC system. A simple dual online ultra-high pressure liquid chromatography system (sDO-UHPLC [18]) was equipped with two analytical RP columns (75 μm × 100 cm, Jupiter, 3 μm, 300 Å, Phenomenex, Torrence, CA) and two solid phase extraction (SPE) columns (150 μm × 3 cm, Jupiter, 3 μm, 300 Å). Solvent A and solvent B were 0.1% formic acid in water and 0.1% formic acid in acetonitrile respectively. A 180-min linear gradient (1–40% solvent B over 160 min, 40–80% over 5 min, 80% for 10 min and holding at 1% for 5 min) and 240 min linear gradient (1–50% solvent B over 220 min, 50–80% over 5 min, 80% for 10 min, and holding at 1% for 5 min) were used for global proteome and phosphoproteome profiling, respectively. The column flow rate was 300 nL/min and the temperature of the column was controlled at 60 °C. Mass spectrometric analysis was performed using a Q Exactive with applied electric potential of electrospray ionization of 2.4 kV and the temperature of the desolvation capillary was set at 250 °C. Full MS scans (400–2000 Th) were acquired at 70,000 resolution with an automated gain control (AGC) target value of 1.0 × 10^6^ and a maximum ion injection time of 20 ms. MS/MS data were acquired using a data-dependent acquisition (DDA) mode and the 10 most abundant ions were isolated with an isolation window of ± 0.8 Th; these ions were fragmented by higher-energy collisional dissociation (HCD) with normalized collision energy of 35 and exclusion duration of 30 s. The MS/MS scans were acquired at the resolution of 17,500 with fixed first mass of 120 Th and the target AGC value and the maximum IT were 1.0 × 10^6^ and 60 ms, respectively. The mass spectrometry proteomics data have been deposited in the ProteomeXchange Consortium via the PRIDE [19] partner repository with the dataset identifier PXD010710 and can be accessed with the following information (Username: reviewer91216@ebi.ac.uk; Password: 7dRc2zNt).

### MaxQuant search

LC-MS/MS mass spectrometry data were searched against a human protein sequence database downloaded from UniProt (20,997 reviewed proteins) using MaxQuant (Max Plank Institute of biochemistry, Andromeda search algorithm) [20]. MaxQuant searches for global proteome analysis were carried out with the following parameters: 4.5 ppm and 20 ppm mass tolerances for precursor and fragments, respectively; fully tryptic peptides with up to two missed cleavages for enzymatic cleavage; peptide N-terminal acetylation and methionine oxidation as variable modifications; cysteine carbamidomethylation as a fixed modification; at least 7 amino acids for the peptide length and less than 4600 Da for the maximum peptide mass; 0.75 for precursor intensity fraction. In the case of phosphoproteome analysis, phosphorylation at serine, threonine, and tyrosine was set as an additional variable modification. Peptides and phosphopeptides were identified by applying 1% false discovery rates at both peptide spectrum match and protein levels. Phosphosite probability was estimated by MaxQuant.

### Unsupervised hierarchical clustering and DAVID functional analysis

Perseus was used to calculate unsupervised hierarchical clustering as described previously [21]. First reporter ion intensities of all phosphopeptides were uploaded onto the Perseus platform and normalized by calculating Z-score values based on each phosphopeptide. To carry out the clustering, the correlation option was used for both raw and column data. Color coding was adjusted for good representation. To search for associated pathways, genes that were specifically associated with one sample relative to the others were clustered. The DAVID [22] bioinformatics resource was used to retrieve enriched pathways based on the resulting lists of genes. To select statistically significant associative pathways, Benjamini correction adjusted p values were applied; i.e., − log (adjusted p value) was at least 3 in at least one sample.

## Results

Clinical samples such as PDAC tissues are often treated with RNAlater for the study of gene expression. Recent advances in mass spectrometry-based proteomics provide proteome profiles that approach the gene coverage of mRNA sequencing, allowing cancer proteogenomic studies [23–25]. It is therefore necessary to explore whether RNAlater influences proteomes and protein modifications such as phosphorylation. In this study, we designed a comprehensive proteome profiling experiment to quantitatively explore the effect of RNAlater on the proteome and phosphoproteome.

### Comprehensive proteome and phosphoproteome profiling of PDAC tissues

**Fig. 1:**
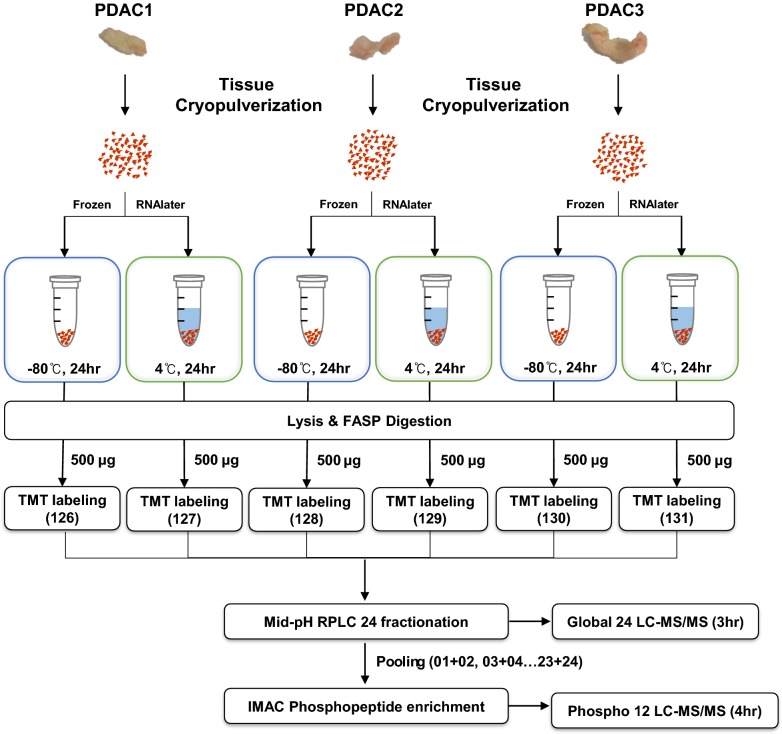
Experimental workflow. Tissues were cryopulverized to prepare frozen powder, of which one portion was treated directly with RNAlater while the other portion was stored at – 80 °C. After 24 h, samples were lysed for FASP digestion. Peptides were labeled with 6-plex TMT reagent, mixed, and then fractionated into 24 fractions for global proteome analysis or 12 fractions for phosphoproteome analysis. Each fraction was analyzed by an Orbitrap mass analyzer

The overall experimental workflow is shown in Fig. 1. Fresh frozen PDAC tissues from three different patients were first cryopulverized to give homogeneous powder samples and then divided into two portions. One portion was stored at – 80 °C (frozen tissue) and the other was treated with RNAlater for 24 h at 4 °C (RNAlater tissue). The frozen tissues and RNAlater tissues shared the same ischemia and tissue content and only differed by RNAlater treatment or no treatment. The tissue powder portions were individually sonicated in the present of lysis buffer containing phosphatase inhibitors. After FASP digestion, peptides were labeled with 6-plex TMT regents, followed by mid-pH RPLC fractionation. Next, 7% of the peptides of each of the 24 mRP fractions were used for global proteome profiling experiments to obtain 24 LC-MS/MS datasets. The remainders of the peptide fractions were concatenated into 12 fractions, which were subjected to separate IMAC phosphopeptide enrichment experiments. The resultant 12 phosphopeptide samples were individually analyzed to obtain 12 LC-MS/MS datasets for phosphoproteome data. A total of 1,997,663 and 1,205,788 tandem mass spectra were collected for the global proteome and phosphoproteome analyses, respectively. A search against a reference protein database using the MaxQuant platform identified 99,136 distinct peptides of 8803 proteins and 16,436 phosphosites of 4550 phosphoproteins at 1% false discovery rates at the levels of both peptide-spectrum matches and proteins.

**Fig. 2:**
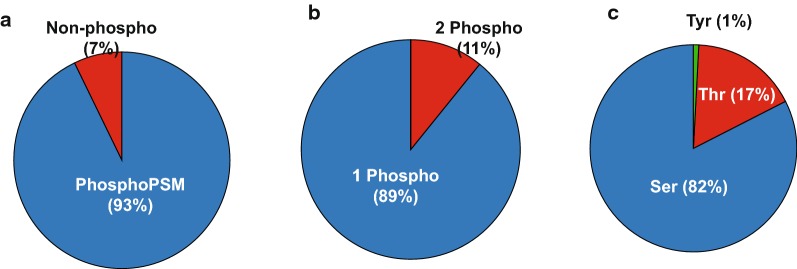
Unbiased and in-depth mass spectrometry result of phosphoproteome. Phosphopeptide enrichment efficiency (**a**) was depicted in pie chart. Based on probability of phosphosites measured, the number of phosphosites per peptide (**b**) and distribution of amino acid residues of phosphopeptides (**c**) were depicted in pie charts

To detect any small but potentially biologically important influences of RNAlater on the proteome, it is important to obtain unbiased and comprehensive quantitative information at the levels of the proteome and phosphoproteome. For this, we employed methodologies such as SEPG [17] to enrich phosphopeptides and sDO-UHPLC [18] to increase the depth and throughputs of the mass spectrometry analysis. Enrichment of phosphopeptides reached > 93% in specificity (Fig. 2a) and more than two thirds of the phosphosites were measured with at least 0.75 site localization probability. As shown in Fig. 2b, almost all phosphopeptides had one or two phosphorylation sites (~ 89% singly phosphorylated peptides and ~ 11% doubly phosphorylated peptides). Phosphosites were mostly at serine residues (~ 82%) although some phosphosites were also detected at threonine (~ 17%) and tyrosine (~ 1%) residues (Fig. 2c). These findings are generally consistent with results of other large-scale phosphoproteomic studies [26].

### Comparison of variations in proteome and phosphoproteome across patient tissues with variations induced by RNAlater

**Fig. 3:**
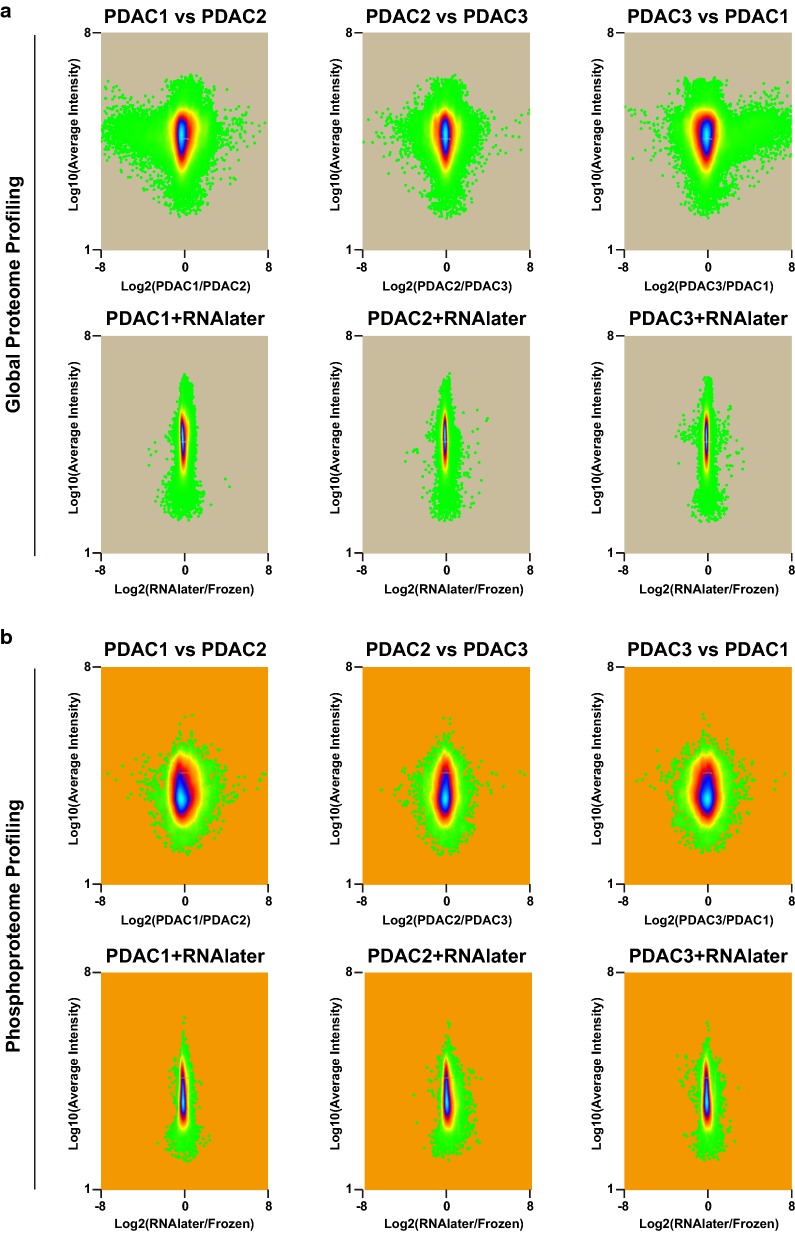
Distributions of differences in reporter ion intensities by RNAlater. Distributions of quantitative differences between RNAlater versus frozen samples and between the three patient samples were depicted using dot gradient plots using the Perseus software. A greater fold change was observed between patients for both proteome (**a**) and phosphoproteome (**b**), whereas only minor changes were induced by RNAlater in all cases

Based on TMT reporter ion intensities of peptides between the frozen and RNAlater tissues, we analyzed the changes (i.e., log2-fold-changes) in both the global proteome and phosphorylation induced by RNAlater and compared them across three patients. As shown in Fig. 3a, the differences in proteins between the frozen and RNAlater tissues (log2(RNAlater/Frozen)) were significantly smaller than the differences observed among patients (i.e., log2(PDAC1/PDAC2)). This result is consistent with previous observations that the global proteome was not affected by RNAlater [11, 12]. Similar results were obtained from our extensive phosphoproteome profiling data (Fig. 3b).

**Fig. 4:**
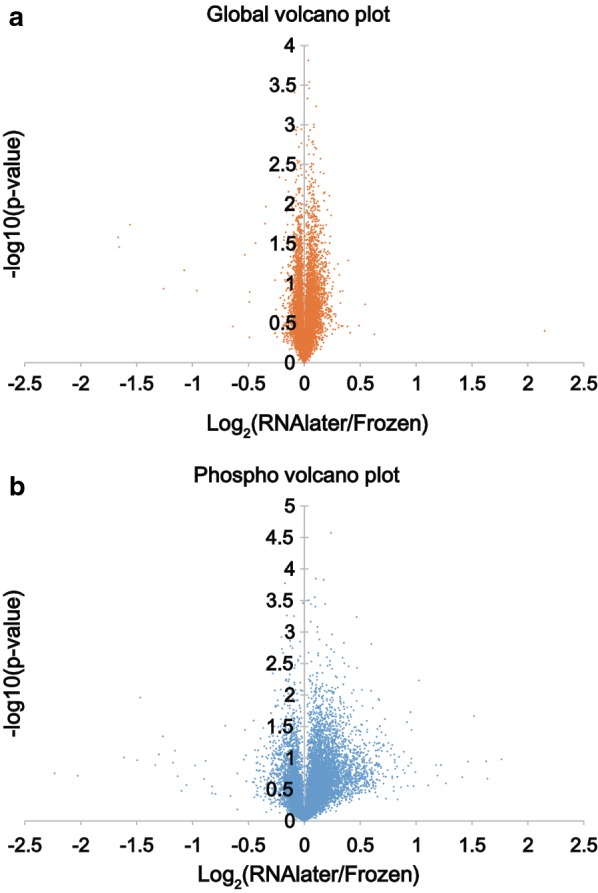
No significant alterations induced by RNAlater. Volcano plots were drawn for the global proteome (**a**) and phosphoproteome (**b**) data. Fold changes induced by RNAlater were calculated and p values were estimated based on Student t test

Nonetheless, to analyze whether some phosphorylation sites show slight alterations while the majority remain unchanged, we generated volcano plots for proteome and phosphoproteome changes induced by RNAlater. As shown in Fig. 4a, b, RNAlater did not induce great changes in either the proteome or the phosphoproteome; the change in the proteome was almost negligible while there was a slight increase in the global level of phosphorylation. Only four phosphopeptides showed statistically significant changes with ≥ 2-fold alteration after RNAlater treatment, as shown in Table 1. Due to the lack of significantly altered phosphorylation, we were not able to carry out pathway analysis.

**Table 1:** Four phosphopeptides found to be significantly altered by RNAlater

Protein ID	Gene symbol	Amino acid	Position within protein	Localization probability	Sequence window
P84101	SERF2	S	21	0.95	QRELARQKNMKKQSDSVKGKRRDDGLSAAAR
P05114	HMGN1	S	89	0.99	LPAENGETKTEESPASDEAGEKEAKSD___
Q7RTV5	AAED1	S	12	1	___MAAPAPVTRQVSGAAALVPAPSGPDSG
Q07002	CDK18	S	12	0.99	___MNKMKNFKRRFSLSVPRTETIEESLAE

These data confirm that tumor samples stored in RNAlater can be used to measure changes in global protein and phosphorylation in tumorigenesis without artificial influences by RNAlater and therefore the RNAlater tissues and fresh frozen tissues should provide very similar, if not identical, proteogenomics results.

### Heterogeneously altered phosphoproteomes among individual pancreatic cancers

**Fig. 5:**
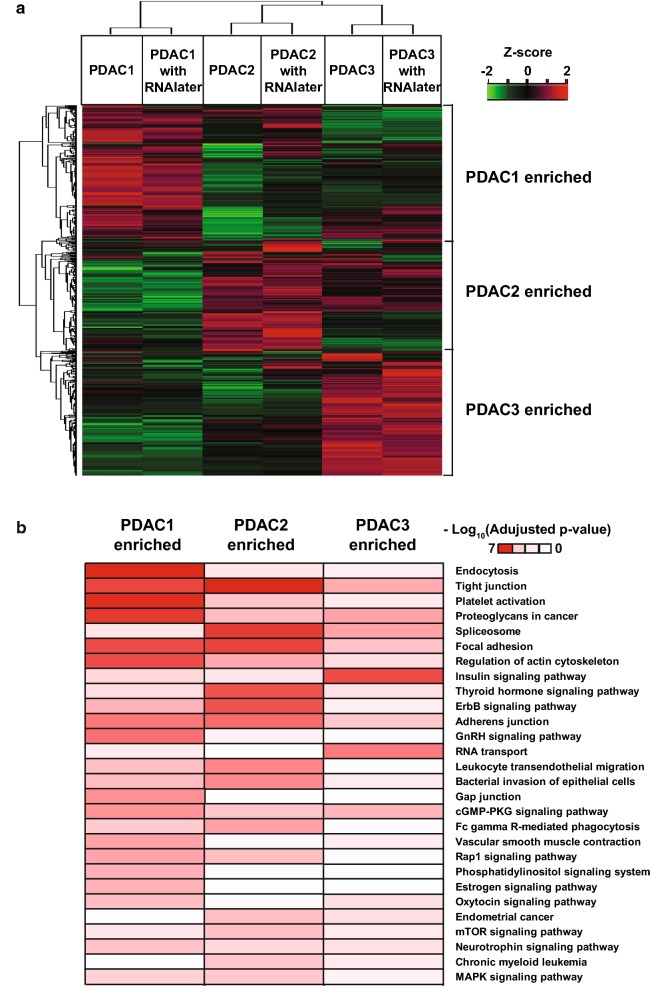
Heterogeneously activated pathways in the three patient samples. **a** Z-score-based unsupervised hierarchical clusterings were carried out using Perseus software and lists of genes found to be enriched in the three patients were generated. **b** Using the lists of genes, patient-specific pathways were identified through DAVID functional analysis. The red color indicates ‘highly activated’ while the whitish color indicates ‘not activated’

We also investigated activated pathways in the three PDAC patients. After normalization, unsupervised hierarchical clustering was carried out to find phosphorylation changes that were specific to each PDAC tissue. As shown in Fig. 5a, the unsupervised clustering resulted in grouping of the patient samples regardless of RNAlater treatment, confirming its negligible effect on the phosphoproteome. Interestingly, all three PDAC samples showed heterogeneous phosphoproteome activation. To identify the associated pathways, we carried out DAVID functional analysis to find KEGG pathways specifically activated in each PDAC sample. As shown in Fig. 5b, multiple pathways were found to be activated in PDAC1, PDAC2, and PDAC3. For example, endocytosis, focal adhesion, and tight junction were highly associated pathways in PDAC1; spliceosome, ErbB signaling, and mTOR signaling pathways were associated with PDAC2; and insulin signaling and RNA transport were associated with PDAC3. This result showed that PDAC is highly heterogeneous and individual patients might need different clinical treatment regimens.

## Conclusions

The proteome and phosphoproteome of tumors represent the status of the disease. DNA and RNA from tumor samples is generally stabilized by RNAlater. Although the effects of RNAlater on biomolecules have been explored, there are no reports to measure its influence on the proteome and phosphoproteome with comprehensive proteome and phosphoproteome data. In this study, we conducted a comprehensive deep quantitative mass spectrometry analysis of the effect of RNAlater on the proteome and phosphoproteome of PDAC. The global proteome and phosphoproteome data indicated no significant changes induced by RNAlater for both proteins and phosphorylations. Further analysis of phosphoproteome data revealed heterogeneously activated pathways among patients, which were not influenced by RNAlater. When we compared this finding with the clinicopathological characteristics of the three patients, the shorter disease free survival may be associated with activated spliceosome and ErbB signaling. Recently introduced proteogenomics technology is being further developed by the integration of multi-omes such as the genome, transcriptome, proteome, and phosphoproteome to understand the biological/clinical state of diseases more thoroughly [27, 28]. This approach has been applied to a multitude of research areas including clinical studies of colorectal cancer, ovarian cancer, and breast cancer [23–25] and basic research studies such as naïve CD4 T cells and B cells [29, 30]. As proteogenomics approaches become more popular, tumor tissues stored in RNAlater may be valuable resources for analyses leading to a thorough molecular understanding of the disease.

## Additional file

**Additional file 1. Table S1.** Clinicopathological features of the three patients.
