# Correction to: Comprehensive proteome and phosphoproteome profiling shows negligible influence of RNAlater on protein abundance and phosphorylation

**DOI:** 10.1186/s12014-019-9247-z

**Published:** 2019-07-11

**Authors:** Jingi Bae, Su-Jin Kim, Seung-Eun Lee, Wooil Kwon, Hongbeom Kim, Youngmin Han, Jin-Young Jang, Min-Sik Kim, Sang-Won Lee

**Affiliations:** 1 Department of Chemistry, Center for Proteogenome Research, Korea University, Seoul, 136-701 Republic of Korea; 2 Department of Biomedical Science and Technology, Kyung Hee Medical Science Research Institute, Kyung Hee University, Seoul, Republic of Korea; 3 Department of Surgery and Cancer Research Institute, Seoul National University College of Medicine, Seoul, Republic of Korea; 4 Department of New Biology, DGIST, Daegu, 42988 Republic of Korea

## Correction to: Clinical Proteomics (2019) 16:18 10.1186/s12014-019-9239-z

In the version of this article that was originally published [1], some information in the “Acknowledgements” section was omitted. This has now been corrected in this Correction note.

## References

[1] Bae J (2019). Clin Proteom.

