# Supplementary material for: Comprehensive proteome and phosphoproteome profiling shows negligible influence of RNAlater on protein abundance and phosphorylation
Source: Clin Proteomics. 2019 Apr 25;16:18. doi: 10.1186/s12014-019-9239-z (PMC6482574; doi:10.1186/s12014-019-9239-z)
Supplement: Supplementary file 1 — Additional file 1. Table S1. Clinicopathological features of the three patients. [file 12014_2019_9239_MOESM1_ESM.docx]

Supplementary Table S1. Clinicopathological features of the three patients

|  | Patient 1 | Patient 2 | Patient 3 |
| --- | --- | --- | --- |
| Gender | Female | Male | Female |
| Age | 56 year | 54 year | 70 year |
| Disease stage | II | II | II |
| Size max | 2.5 cm | 3.1 cm | 3.1 cm |
| Disease free survival | 3 month | 1 month | 3 month |
